# Effects of two different anesthesia-analgesia methods on incidence of postoperative delirium in elderly patients undergoing major thoracic and abdominal surgery: study rationale and protocol for a multicenter randomized controlled trial

**DOI:** 10.1186/s12871-015-0118-5

**Published:** 2015-10-13

**Authors:** Ya-Wei Li, Hui-Juan Li, Huai-Jin Li, Yi Feng, Yao Yu, Xiang-Yang Guo, Yan Li, Bin-Jiang Zhao, Xiao-Yun Hu, Ming-Zhang Zuo, Hong-Ye Zhang, Mei-Rong Wang, Ping Ji, Xiao-Yan Yan, Yang-Feng Wu, Dong-Xin Wang

**Affiliations:** 1 Department of Anesthesiology and Critical Care Medicine, Peking University First Hospital, No.8 Xishiku Street, Xicheng District, Beijing, 100034 China; 2 Peking University Clinical Research Institute, No.38 Xueyuan Road, Haidian District, Beijing, 100191 China; 3 Department of Anesthesiology, Peking University People’s Hospital, No.11 Xizhimen South Street, Xicheng District, Beijing, 100044 China; 4 Department of Anesthesiology, Peking University Third Hospital, No.49 Huayuan North Road, Haidian District, Beijing, 100191 China; 5 Department of Anesthesiology, Beijing Shijitan Hospital, No.10 Tieyi Road, Haidian District, Beijing, 100038 China; 6 Department of Anesthesiology, Beijing Hospital, No.1 Dahua Road, Dongcheng District, Beijing, 100730 China

**Keywords:** Aged, Surgical procedures, Anesthesia, Epidural, Analgesia, Epidural, Postoperative complications, Delirium

## Abstract

**Background:**

Delirium is a common complication in elderly patients after surgery and associated with increased morbidity and mortality. Studies suggest that deep anesthesia and intense pain are important precipitating factors of postoperative delirium. Neuraxial block is frequently used in combination with general anesthesia for patients undergoing major thoracic and abdominal surgery. Compared with general anesthesia alone and postoperative intravenous analgesia, combined epidural-general anesthesia and postoperative epidural analgesia decreases the requirement of general anesthetics during surgery and provided better pain relief after surgery. However, whether combined epidural-general anesthesia plus epidural analgesia is superior to general anesthesia plus intravenous analgesia in decreasing the incidence of postoperative delirium remains unknown.

**Methods/design:**

This is a multicenter, open-label, randomized, parallel-controlled clinical trial. One thousand eight hundred elderly patients (age range 60–90 years) who are scheduled to undergo major thoracic or abdominal surgery are randomized to receive either general anesthesia plus postoperative intravenous analgesia or combined epidural-general anesthesia plus postoperative epidural analgesia. The primary outcome is the 7-day incidence of postoperative delirium. Secondary outcomes include the duration of postoperative delirium, the intensity of pain during the first three days after surgery, the 30-day incidences of postoperative non-delirium complications, the length of stay in hospital after surgery and 30-day all-cause mortality.

**Discussion:**

Results of the present study will provide information to guide clinical practice in choosing appropriate anesthesia-analgesia method for elderly patients undergoing major thoracic and abdominal surgery.

**Trial registration:**

The study is registered on ClinicalTrials.gov NCT01661907 and Chinese Clinical Trial Registry ChiCTR-TRC-12002371.

## Background

Delirium is an acute mental syndrome characterized by (1) disturbance of consciousness with reduced ability to focus, sustain and shift attention, (2) change in cognition (such as memory deficit, disorientation, or language disturbance) or development of a perceptual disturbance that is not better accounted for by a preexisting, established or evolving dementia, and (3) disturbance developing over a short period of time (usually hours to days) and tending to fluctuate during the course of the day [1].

Postoperative delirium is a common complication in the elderly after surgery. The reported incidences vary widely (from 0 % to 73.5 %) with an overall incidence of 36.8 %, and the incidence increases with age [2]. In the sickest patients in the intensive care unit (ICU), the incidence can be up to 80 % [3]. In our previous studies, delirium occurred in 51 % of patients after cardiac surgery [4] and in 44.5 % of those after non-cardiac surgery [5]. The occurrence of postoperative delirium is associated with worse outcomes, including prolonged length of stay in the ICU and hospital, increased morbidity and mortality, compromised long-term cognitive function and physical ability, and elevated medical care costs [6–15]. The most effective way to minimize such adverse outcomes is the prevention of its occurrence. At present, however, strategies that can effectively prevent the occurrence of postoperative delirium are limited [16, 17].

The causes of postoperative delirium are multifactorial and include predisposing and precipitating factors [18]. Studies found that deep anesthesia and intense postoperative pain are important precipitating factors [17, 19]. In a retrospective cohort study of patients undergoing aortic surgery, deep anesthesia (bispectral index reduction > 25 % from baseline) was associated with high incidences of postoperative delirium and neurological events [20]. In a randomized controlled trial of patients undergoing minor ophthalmic surgery, auditory evoked potential-guided anesthesia reduced the requirement of anesthetic agents and relieved early postoperative cognitive decline [21]. In two randomized controlled trials of elderly patients undergoing major noncardiac surgery, bispectral index-guided general anesthesia reduced anesthetic exposure and episodes of deep anesthesia, and decreased the incidences of postoperative delirium [22, 23].

Opioid is the mainstay of postoperative analgesia. However, high-dose opioids are associated with an increased risk of postoperative delirium [24, 25]. In a pilot randomized clinical trial, use of gabapentin as an add-on agent of postoperative analgesia reduced the occurrence of postoperative delirium, possibly due to its opioid-sparing effect [26]. In a prospective observational study of elderly patients undergoing total hip or knee arthroplasty, supplemental use of opioid-sparing drugs (gabapentin, paracetamol, and celecoxib) for postoperative analgesia was associated with a reduced incidence of delirium [27].

Neuraxial anesthesia and analgesia are used in patients undergoing lower abdominal or lower extremity surgery. When compared with general anesthesia and intravenous analgesia, neuraxial anesthesia and analgesia provides more postoperative benefits, including better pain relief, shortened length of stay in the ICU and hospital, and reduced morbidity and mortality [28–33]. Meta-analyses showed that regional anesthesia decreases the risk of postoperative cognitive dysfunction, but not delirium, when compared to general anesthesia [34, 35].

For patients undergoing major thoracic and abdominal surgery, neuraxial block can be performed in combination with general anesthesia. Studies showed that, compared with general anesthesia and postoperative intravenous analgesia, combined epidural-general anesthesia and postoperative epidural analgesia decreased the requirement of general anesthetics during surgery and provided better pain relief after surgery [36–39]. We hypothesize that combined epidural-general anesthesia and epidural analgesia may be superior to general anesthesia and intravenous analgesia in decreasing the incidence of postoperative delirium. So far, this effect has not been evaluated in previous studies [40–42].

The purpose of this study is to investigate whether two different anesthesia-analgesia methods (combined epidural-general anesthesia plus postoperative epidural analgesia versus general anesthesia plus postoperative intravenous analgesia) would lead to a difference in the incidence of postoperative delirium in elderly patients after major thoracic and abdominal surgery.

## Methods/design

### Study design

This is a multicenter, open-label, randomized controlled clinical trial with two parallel arms, combined epidural-general anesthesia plus postoperative epidural analgesia group (EGA group) and general anesthesia plus postoperative intravenous analgesia group (GA group). A total of 1800 patients undergoing major thoracic and abdominal surgery will be recruited, with 900 patients for each group. The study is coordinated by the Department of Anesthesiology and Critical Care Medicine of Peking University First Hospital, and Peking University Clinical Research Institute is responsible for the study monitoring, data management and data analysis. Other centers participating in the study include Peking University People’s Hospital, Peking University Third Hospital, Beijing Hospital, and Beijing Shijitan Hospital, all of which are teaching hospitals of Peking University.

### Ethics approval

The study protocol has been approved by the Peking University Institutional Review Board (IRB) (IRB00001052-11048). Any protocol modifications will be submitted for the IRB review and approval. The study has been registered at ClinicalTrials.gov (NCT01661907) and Chinese Clinical Trial Registry (ChiCTR-TRC-12002371). Written informed consent will be obtained from each patient or, if the patient cannot provide informed consent, from the surrogate of the patient.

### Participants

The inclusion criteria are (1) elderly patients (age range 60–90 years), (2) scheduled to undergo open thoracic or open abdominal surgery with an expected duration of 2 hours or longer. For those who undergo thoracoscopic or laparoscopic surgery, the expected length of incision must be 5 centimeters or more, and (3) agree to receive patient-controlled postoperative analgesia.

Patients who meet any of the following criteria will be excluded: (1) previous history of schizophrenia, epilepsy or Parkinson disease, or unable to complete preoperative assessment due to severe dementia, language barrier or end-stage disease; (2) history of myocardial infarction within 3 months before surgery; (3) any contraindication to epidural anesthesia and analgesia, including abnormal vertebral anatomy, previous spinal trauma or surgery, severe chronic back pain, coagulation disorder (prothrombin time or activated partial prothrombin time longer than 1.5 times of the upper limit of normal, or platelet count of less than 80 × 10^9^/L), local infection near the site of puncture, and severe sepsis; (4) severe heart dysfunction (New York Heart Association functional classification 3 or above), hepatic insufficiency (Child-Pugh grades C), or renal insufficiency (serum creatinine of 442 μmol/L or above, with or without serum potassium of 6.5 mmol/L or above, or requirement of renal replacement therapy); or (5) any other conditions that were considered unsuitable for study participation.

### Personnel training and delirium assessment

Prior to the study, study personnel are trained to follow the study protocol according to the Good Clinical Practice principles. They are also trained by a psychiatrist to assess delirium with the Confusion Assessment Method for the Intensive Care Unit (CAM-ICU) which is performed in two steps [43, 44]. First, level of arousal/agitation will be assessed with the Richmond Agitation Sedation Scale (RASS) [45, 46]. This is a 10-point scale with four levels of anxiety or agitation (+1 restless to +4 combative), one level of alert and calm (0), and five levels of sedation or coma (−1 lethargy to −5 nonarousable). Second, delirium is diagnosed by the CAM-ICU. It detects four features of delirium: (a) acute onset of mental status changes or a fluctuating course, (b) inattention, (c) disorganized thinking, and (d) altered level of consciousness. Delirium is defined as a RASS score of −3 or higher, with features of both (a) and (b), accompanied by either (c) or (d). Coma is defined as a RASS score of −4 or −5 where CAM-ICU cannot be assessed.

Delirium episodes are classified into three motoric subtypes. Hyperactive delirium means that the RASS scores are persistently positive (+1 to +4) during every CAM-ICU positive assessment; hypoactive delirium means that the RASS scores are persistently neutral or negative (between 0 and −3) during every CAM-ICU positive assessment; and mixed delirium means that some RASS scores are positive (+1 to +4) whereas some RASS scores are neutral or negative (between 0 and −3) during every CAM-ICU positive assessment [47, 48].

### Patient recruitment and baseline data collection

The day before surgery (or Friday for those who will undergo surgery on next Monday), investigators who are authorized by the principal investigator will check the list of patients planned for surgery and their medical charts to identify potential participants according to our inclusion and exclusion criteria. They will then visit these patients to formally invite them for participation.

For patients who meet the inclusion/exclusion criteria and give written informed consents, baseline data including demographic data, preoperative diagnosis, medical history, medication history, surgical history, and main results of physical, laboratory, and instrumental examinations will be collected. The following preoperative evaluation will also be performed, i.e., the activities of daily living are assessed with Barthel Index [49, 50], cognitive function is assessed with the Mini-Mental State Examination [51, 52], levels of anxiety and depression are assessed with the Hospital Anxiety and Depression Scale [53, 54], delirium is assessed with the CAM-ICU [43, 44].

### Randomization

**Fig. 1:**
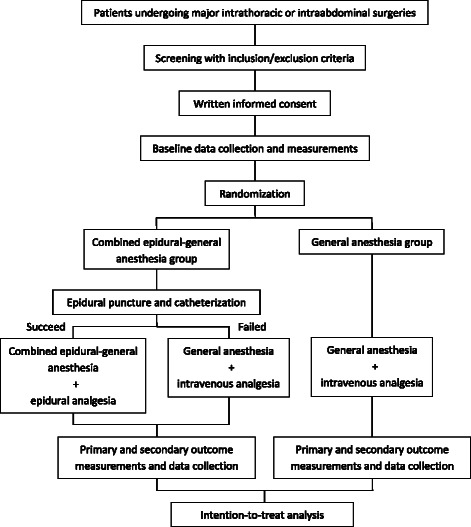
Design of the study

The randomization will be performed centrally at Peking University Clinical Research Institute through an interactive web response system (IWRS, Brightech Clinical Information Management System) on the day of surgery just before anesthesia. The randomization is stratified by medical center and type of surgery (thoracic or abdominal surgery) (Fig. 1).

### Anesthesia and postoperative analgesia

For patients of both groups, no premedication is administered; dexmedetomidine is not allowed; anticholinergics are prohibited unless being used for the treatment of bradycardia, in which case atropine will be administered. Intraoperative monitoring includes electrocardiogram, non-invasive blood pressure, pulse oxygen saturation, end-tidal concentrations of inhalational anesthetics and carbon dioxide, nasopharyngeal temperature, and urine output. Radial arterial pressure and central venous pressure are monitored when necessary.

#### General Anesthesia plus Postoperative Intravenous Analgesia (GA Group)

For patients assigned to receive general anesthesia plus postoperative intravenous analgesia (GA Group), anesthesia will be induced with midazolam (0.02-0.03 mg/kg), propofol, sufentanil and rocuronium. For patients with expected difficult airway, endotracheal intubation may be facilitated by succinylcholine or awake intubation may be performed. Anesthesia will be maintained with either intravenous (propofol), inhalational (sevoflurane with or without nitrous oxide), or combined intravenous-inhalational anesthetics. Additional opioids (remifentanil, sufentanil, fentanyl, or morphine) and muscle relaxant (rocuronium, atracurium, or cisatracurium) will be administered when deemed necessary by the attending anesthesiologists.

Patient-controlled intravenous analgesia will be provided after surgery. This is established with 50 mg morphine in 100 mL normal saline, programmed to deliver a 2-mL bolus with a 6–10 minutes lockout interval and a 1 mL/hr background infusion. For patients with low body weight or poor general condition, doses can be decreased and upper dose limit can be set for the patient-controlled pump.

#### Combined Epidural-General Anesthesia plus Postoperative Epidural Analgesia (EGA Group)

For patients assigned to receive combined epidural-general anesthesia plus postoperative epidural analgesia (EGA Group), epidural catheterization will be performed first. The intervertebral space for epidural puncture will be selected by the attending anesthesiologists according to the site of planned incision. An epidural catheter will be inserted using a standard technique. After negative aspiration for blood and cerebrospinal fluid, a test dose of 3–4 mL of 2 % lidocaine will be administered to confirm the position of the catheter.

General anesthesia will also be induced with midazolam (0.02-0.03 mg/kg), propofol, sufentanil and rocuronium. Anesthesia will be maintained with intravenous (propofol), inhalational (sevoflurane with or without nitrous oxide), or combined intravenous-inhalational anesthetics, together with 0.375 %–0.5 % ropivacaine administered bolusly and/or continuously through the epidural catheter. Additional opioids (remifentanil, sufentanil, fentanyl, or morphine) and muscle relaxant (rocuronium, atracurium, or cisatracurium) will be administered when deemed necessary.

Patient-controlled epidural analgesia will be provided after surgery. This is established with 0.12 % ropivacaine and 0.5 μg/mL sufentanil in 250 mL normal saline, programmed to deliver a 2-mL bolus with a lockout interval of 20 minutes and a background infusion of 4 mL/hr. For patients with low body weight or poor general condition, doses can be decreased and upper dose limit can be set for the patient-controlled pump.

All anesthesiologists participating in the study will be experienced in epidural block techniques. But even in experienced hands, we can anticipate a failure rate of about 5 %. Patients with unsuccessful epidural blocks will receive general anesthesia and postoperative intravenous analgesia as that for the GA Group. However, data of these patients will be included in the EGA Group in the final intention-to-treat analysis (Fig. 1).

### Follow-up schedule and outcome assessment

Patients will be visited twice daily during the first seven days after surgery for assessment of delirium and monitoring of non-delirium complications. They will then be followed up weekly until 30 days after surgery for monitoring of non-delirium complications and assessment of outcome. Detailed perioperative data including duration of anesthesia, doses of sedatives, anesthetics and analgesics used during anesthesia, type and duration of surgery, intraoperative fluid balance and transfusion of blood products, use of drugs with anticholinergic properties as well as postoperative analgesic consumption will be collected.

For patients who develop delirium, management will be administered according to routine practice, i.e., nonpharmacologic treatment will be applied first, pharmacologic treatment will be reserved only for those with severe agitation. Meanwhile, potential causes will be searched and corrected [55, 56].

#### Primary outcome

The primary outcome is the 7-day incidence of postoperative delirium. Patients will be assessed for delirium with the CAM-ICU twice daily (from 8 to 10 a.m. and from 6 to 8 p.m.) during the first seven days after surgery. Study personnel who assess delirium do not participate in intraoperative anesthesia and postoperative management of patients.

#### Secondary outcomes

Secondary outcomes include the duration of postoperative delirium, the intensity of pain during the first three days after surgery, the occurrence of non-delirium complications within 30 days after surgery, the length of stay in hospital after surgery, and the 30-day all-cause mortality.

The intensity of postoperative pain both at rest and with coughing will be evaluated twice daily at the same time of delirium assessment (i.e., from 8 to 10 a.m. and from 6 to 8 p.m.) with the numeric rating scale (NRS, a 11-point scale where 0 indicates no pain and 10 indicates the worst pain). For patients who are deeply sedated or unarousable (−4 or −5 on the RASS), pain evaluation will be stopped temporarily and repeated later. Analgesic consumption will be recorded daily during the first three days after surgery. Non-delirium complications are defined as medical conditions other than delirium that required therapeutic intervention.

### Adverse events

An adverse event indicates any unpredictable, unfavorable medical event that is associated with any medical intervention and occurs during the study period. It can be related to the study intervention or otherwise. All adverse events will be monitored carefully, managed promptly according to routine practice, and followed up until they are properly resolved, stabilized or recovered to normal. The information including the type, date(s) of onset and resolution (if applicable), severity of influence, relationship with grouping, treatment and outcome (sequelae) will be recorded in the Case Report Forms. They will be reported to the IRB in the final report.

A severe adverse event indicates any unpredictable medical events that lead to death, threat of life, prolonged length of hospital stay, persistent disability or dysfunction, or other severe event. All severe adverse events will be monitored, managed, followed up and recorded as above, and will be reported to the IRB within 24 hours after their occurrence.

### Sample size estimation

In a recent cohort study of our own, the incidence of postoperative delirium in elderly patients after major abdominal surgery (performed under general anesthesia followed by intravenous analgesia) was 13.1 %. In our previous study, the incidence of delirium was reduced by roughly 1/3rd when the intervention (haloperidol prophylaxis) was administered in elderly patients after noncardiac surgery [57]. Assuming that the GA Group (general anesthesia plus postoperative intravenous analgesia) in the present study will have a similar delirium incidence as in our previous study, a total of 1664 subjects (832 subjects in each group) are required to detect a 30 % reduction in the incidence of postoperative delirium at a 90 % power with a two-sided significance level of 0.05. Considering a dropout rate of about 7.5 %, we plan to enroll 1800 patients during a 3-year period.

### Data analysis

All statistical analyses will be performed with SAS statistical package, version 9.3 (SAS Institute, Cary, NC, USA) by statisticians at Peking University Clinical Research Institute. Analyses will be done on an intention-to-treat basis, that all subjects will be analyzed in the group which they were assigned to.

Primary outcome (the 7-day incidence of postoperative delirium) will be analyzed using Cochran-Mantel-Haensel Chi-squared tests, stratified by medical center and type of surgery (thoracic or abdominal surgery). The 95 % confidence interval of the incidence of each group and the difference between the two groups will be calculated. Logistic regression will be used for further analysis if an adequate number of events are observed or any impact or relative factor is expected according to the results of single-factor analysis. For the secondary outcomes, continuous variables (the dose of medications, the NRS pain scores after surgery) will be analyzed using an unpaired T-test, Mann–Whitney U test or repeated-measure analysis of variance; categorical variables (the incidences of non-delirium complications, the daily prevalence of delirium, and the 30-day mortality rate) will be analyzed using the Chi-squared test, continuity correction Chi-squared test or Fisher exact test; time-to-event results (time to onset of delirium or non-delirium complications, duration of hospitalization after surgery) will be analyzed using the Kaplan-Meier estimator, and the differences between groups will be tested by the log-rank test. Also the Cox proportional hazards model will be used to estimate the overall survival HR and 95 % confidence interval.

## Discussion

This multicenter, open-label, randomized, parallel-controlled clinical trial is designed to test the hypothesis that, compared with general anesthesia plus postoperative intravenous analgesia, combined epidural-general anesthesia plus postoperative epidural analgesia reduces the incidence of delirium in elderly patients during the first seven days after major thoracic and abdominal surgery.

Epidural anesthesia and analgesia is widely used for patients undergoing major surgery in combination with general anesthesia. In a large population-based retrospective cohort study of 259037 patients who underwent intermediate-to-high risk non-cardiac surgery between 1994 and 2004, 22 % received epidural anesthesia [58]. In another population-based historical cohort study of 29743 patients who underwent elective major surgery between 2004 and 2011, 27 % received combined general and neuraxial anesthesia [59]. Previous studies showed that, when compared with general anesthesia and opioid analgesia, epidural anesthesia and analgesia decreases the risk of pneumonia, improves the recovery of gastrointestinal function, and reduces the 0-to-30-day mortality after major surgery [28, 33, 60, 61]. However, the impact of the type of anesthesia on the incidence of postoperative delirium was only compared between regional and general anesthesia [40–42].

In the present study, a multicenter design is adopted so that patients enrolled in the study include a variety of surgical procedures under different clinical environments. This will increase the generalizability of our results. Because of the apparent difference between the two anesthesia-analgesia methods, a double-blind design cannot be achieved. Therefore, we had to use an open-label design. The following measures will be undertaken to decrease the risk of potential bias. Firstly, we use central randomization. Secondly, we require that all study personnel who are in charge of postoperative delirium assessment are not involved in intraoperative anesthesia and postoperative management of the enrolled patients, and thus they have no interest conflicts with the results of the study. Thirdly, all study personnel are trained to follow a standard procedure for the preoperative visit and postoperative follow-up and assessment.

Both the Confusion Assessment Method (CAM) [62, 63] and the CAM-ICU [43, 44] can be used for delirium assessment with sufficient sensitivity and specificity and have validated Chinese versions [64, 65]. The former is suitable for non-intubated patients, the later can be used for both intubated and non-intubated patients. In the present study, although the majority of our patients will not be admitted to the ICU with endotracheal intubation, we chose to use the CAM-ICU because of the following reasons. Firstly, the level of sedation/agitation will be assessed and the subtype of delirium (hypoactive, hyperactive, or mixed) can be classified with the use of CAM-ICU [47, 48]. Secondly, the use of a same assessment method for patients with and without endotracheal intubation may be helpful to avoid the potential bias. Thirdly, our team members are more familiar with the CAM-ICU because of previous studies [4, 57].

Strengths of the protocol include that it is the first multicenter, large sample size and centrally randomized study and that it observes both the primary (the 7-day incidence of postoperative delirium) and secondary endpoints (duration of postoperative delirium, incidences of non-delirium complications, length of stay in hospital after surgery, all-cause 30-day mortality, etc.). Results of the study will provide evidence for chosing appropriate method of anesthesia and analgesia in elderly patients undergoing major thoracic and abdominal surgery.

## Trial status

The trial is currently at the stage of patient recruitment and data collection.

## Abbreviations

EGA group: Combined epidural-general anesthesia plus postoperative epidural analgesia group
GA group: General anesthesia plus postoperative intravenous analgesia group
CAM-ICU: Confusion Assessment Method for the Intensive Care Unit
RASS: Richmond Agitation Sedation Scale
NRS: Numeric rating scale
CAM: Confusion assessment method
